# Functional magnetic resonance imaging study during resting state and visual oddball task in mild cognitive impairment

**DOI:** 10.1111/cns.14371

**Published:** 2023-07-20

**Authors:** Kerem Kemik, Emel Ada, Berrin Çavuşoğlu, Cansu Aykaç, Derya Durusu Emek‐Savaş, Görsev Yener

**Affiliations:** ^1^ Department of Neuroscience Institute of Health Sciences, Dokuz Eylül University Izmir Turkey; ^2^ Department of Radiology Dokuz Eylül University Medicine Faculty Izmir Turkey; ^3^ Department of Medical Physics Institute of Health Sciences, Dokuz Eylül University Izmir Turkey; ^4^ Department of Psychology, Faculty of Literature Dokuz Eylül University Izmir Turkey; ^5^ Department of Neurology, Faculty of Medicine Izmir Economy University İzmir Turkey

**Keywords:** fMRI, independent component analysis, mild cognitive impairment, resting‐state, task‐based, visual network

## Abstract

**Background:**

Amnestic mild cognitive impairment (aMCI) is a transitional state between normal aging and dementia, and identifying early biomarkers is crucial for disease detection and intervention. Functional magnetic resonance imaging (fMRI) has the potential to identify changes in neural activity in MCI.

**Methods:**

We investigated neural activity changes in the visual network of the aMCI patients (n:20) and healthy persons (n:17) using resting‐state fMRI and visual oddball task fMRI. We used independent component analysis to identify regions of interest and compared the activity between groups using a false discovery rate correction.

**Results:**

Resting‐state fMRI revealed increased activity in the areas that have functional connectivity with the visual network, including the right superior and inferior lateral occipital cortex, the right angular gyrus and the temporo‐occipital part of the right middle temporal gyrus (p‐FDR = 0.008) and decreased activity in the bilateral thalamus and caudate nuclei, which are part of the frontoparietal network in the aMCI group (p‐FDR = 0.002). In the visual oddball task fMRI, decreased activity was found in the right frontal pole, the right frontal orbital cortex, the left superior parietal lobule, the right postcentral gyrus, the right posterior part of the supramarginal gyrus, the right superior part of the lateral occipital cortex, and the right angular gyrus in the aMCI group.

**Conclusion:**

Our results suggest the alterations in the visual network are present in aMCI patients, both during resting‐state and task‐based fMRI. These changes may represent early biomarkers of aMCI and highlight the importance of assessing visual processing in cognitive impairment. However, future studies with larger sample sizes and longitudinal designs are needed to confirm these findings.

## Graphical abstract

Our study investigated neural activity changes in the visual network of mild cognitive impairment (MCI) patients using resting‐state and task‐based fMRI. We found that alterations in the visual network may represent early biomarkers of MCI and highlight the importance of assessing visual processing in cognitive impairment.

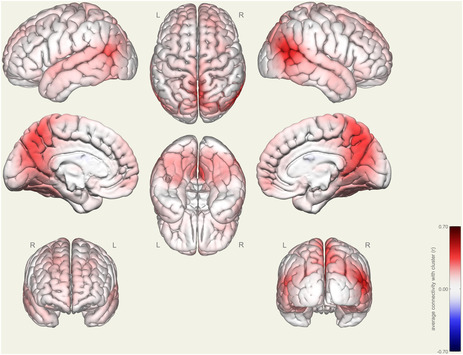

## 1 INTRODUCTION

Mild cognitive impairment (MCI) is a condition characterized by a decline in cognitive functioning that is more significant than normal age‐related cognitive changes but not severe enough to meet the criteria for dementia. It is estimated that 15%–20% of individuals aged 65 years or older have MCI, and these individuals have a higher risk of developing Alzheimer's disease (AD) compared to those without MCI.^1^ As the global population ages, the prevalence of MCI is expected to increase, making it an important public health concern.

Alzheimer's disease is the most common cause of dementia and is characterized by the progressive degeneration of brain cells, leading to memory loss, language difficulties, and other cognitive and behavioral changes. The exact cause of AD is not fully understood, but it is thought to be caused by a combination of genetic, environmental, and lifestyle factors. AD is a major public health challenge, with an estimated 50 million people worldwide currently living with the disease according to the World Health Organization. Amnestic Mild Cognitive Impairment (aMCI) is a transitional stage between normal aging and Alzheimer's disease (AD), characterized by memory impairment beyond what is expected for age and education. It is estimated that 5%–15% of aMCI individuals progress to AD annually, making early detection and intervention critical. In recent years, neuroimaging techniques, such as magnetic resonance imaging (MRI) and functional MRI (fMRI), have emerged as promising tools to identify early biomarkers of aMCI. Studies have shown that aMCI patients have structural and functional changes in the hippocampus, parahippocampal gyrus, and other regions involved in memory and learning.^2^

Studies have focused on identifying biomarkers of cognitive decline, including changes in brain function and connectivity. Functional connectivity refers to the work of different brain regions together in a coordinated manner and is thought to be disrupted in aMCI and AD.^3^ Independent component analysis (ICA) is a data‐driven method that has been used in functional connectivity analysis to identify patterns of neural activity that are consistent across a group of individuals.^4^

Independent Component Analysis (ICA) offers a compelling alternative for analyzing group task fMRI data, providing numerous advantages over traditional ROI‐based methods. By unbiasedly decomposing fMRI data into independent components, ICA allows for a comprehensive exploration of functional connectivity, capturing both known and novel functional networks. ICA facilitates group‐level analyses by enabling the extraction of individual subject‐level components that can be aggregated across participants. This approach ensures the robustness and reliability of the findings, allowing for the identification of shared network architectures that characterize task‐related functional connectivity. Its ability to separate signal from noise, along with its suitability for group‐level analyses, enhances its utility in unraveling the complex interplay of brain regions during task‐based fMRI experiments. The widespread adoption of ICA as the preferred method for investigating functional connectivity in group task fMRI studies holds immense potential for advancing our understanding of cognitive processes and neurologic disorders.^5^, ^6^, ^7^ The visual oddball task is a cognitive task that has been used to investigate attention and working memory processes in the brain. It involves presenting a series of visual stimuli to participants, with occasional “oddball” stimuli that differ from the norm.

In 2010 Babiloni et al defined that the task fMRI was found effective for identifying changes in neural activity associated with aMCI and AD.^8^ Also, recent studies (Erhardt et al in 2011 and Duff et al in 2012) show the analysis of task fMRI findings with ICA is more reliable to investigate the changes in functional connectivity.^9^, ^10^ These changes in functional connectivity were associated with cognitive performance during tasks, suggesting cognitive decline.^11^, ^12^ MCI manifests in different individuals, making it difficult to identify consistent markers of cognitive decline which is why we need to identify specific regions with group analysis.

In this study, our aim was to investigate changes in functional connectivity in resting state fMRI and visual oddball task fMRI findings to help in diagnosis in MCI patients.

## 2 MATERIALS AND METHODS

### 2.1 Participants

The present study was approved by the University Ethics Committee, and written informed consent was obtained from all participants. In this study, a total of 40 participants, 20 of whom were diagnosed with amnestic mild amnestic cognitive impairment (aMCI) by the Dementia Clinic of the Department of Neurology of the Faculty of Medicine, and 20 of whom were healthy controls recruited from the community. Three healthy controls were excluded from the analysis due to poor image quality following MRI scanning, resulting in a final sample of 17 healthy controls and 20 aMCI patients. The two groups were matched in terms of age, and education (Table 1). Shapiro Wilk normality analyses tests are used for the analysis of data distribution and q plots added in the Appendix S1. Participants who scored 12 or more on the geriatric depression scale, used drugs that actively affect the central nervous system, had a history of stroke or trauma, and have epilepsy or psychiatric diseases that affect cognitive functions were not included in the study.

**TABLE 1:** Sociodemographic Factors of Research.

	Control	MCI	*p*
Sex (M/F)	6/11	10/10	0.368^b^
Age	69.6 ± 6.9	69.5 ± 6.6	0.853^a^
Education	10.8 ± 4.0	11.4 ± 3.9	0.375^a^
MMSE scores	29.1 ± 0.8	25.3 ± 1.1	<0.001^a^, *

Abbreviations: M/F, Male/female; MCI, mild cognitive impairment; MMSE, Mini Mental State Exam.

^a^ Mann–Whitney *U* test.

^b^ Chi‐square test.

* *p* < 0.05.

### 2.2 Clinical evaluation

Clinical evaluations were performed on all participants using the Mini Mental State Test (MMSE),^13^ the Clinical Dementia Staging Scale,^14^ and the Geriatric Depression Scale. Individuals with an MMSE score of 28–30 and clinical dementia staging scale of 0 were considered healthy, while individuals with an MMSE score of 24–28 and clinical dementia staging scale of 0.5 were evaluated as MCI. All sociodemographic data are shown in Table 1.

### 2.3 Image acquisition

All participants were evaluated using a 1.5 T MR unit (Achieva Philips Medical Systems, Best, The Netherlands). For anatomical reference, a T1‐weighted anatomical inversion‐recovery scan (TR:2494 ms, TE:15 ms, TI: 350 ms, flip angle: 90, ETL: 5, matrix: 512 × 512, slice thickness: 4 mm) and for functional magnetic resonance imaging (fMRI) T2*‐weighted gradient echo‐planar images (TR: 3000 ms, TE: 50 ms, flip angle: 90° FOV: 230 mm, RFOV: 100%, slice thickness: 4 mm, gap: 0 mm, matrix: 64 × 64, ETL: 48 NA: 1, in‐plane resolution with 28 slices) were obtained. The first five scans were excluded from the analysis to reduce magnetic saturation effects. Two hundred dynamics were acquired for each task. The active and passive volume rates were 12/8 in each cycle of 20 dynamics to reduce the test time. In the resting state, the phase did not use active/passive flags and the resting state was taken before all task‐based fMRIs.

### 2.4 Visual oddball task

The visual oddball task^15^ was used to assess the cognitive function of the participants. During the task, two stimuli (a target stimulus and a standard stimulus) were presented to the participants, and they were asked to count the target stimuli. 10 cd/cm^2^ standard stimulus, 40 cd/cm^2^ target stimulus, and black screen during waiting times were arranged. The target/stimuli rate was one‐third. The image was transferred to the room with a monitor placed in the MRI room, and then this image was transferred to the subject, helping with a mirror placed on the head coil.

### 2.5 fMRI pre‐processing

The fMRI data were preprocessed using the SPM12 software along with the CONN toolbox.^4^ The standard pre‐processing pipeline of CONN 17.0 was employed, which automated most of the pre‐processing steps. This preprocessing included realignment and unwarping, slice‐time correction, art‐based outlier detection, structural segmentation and normalization, functional normalization, outlier detection, and smoothing with an 8 mm Gaussian kernel. The Gaussian kernel was chosen for improving signal noise ratio and improving the validity of the statistical tests by making the error distribution more normal. The kernel uses Full Width Half Maximum (FWHM) procedure. The value of the FWHM rate is recommended to 2× of the slice thickness.^16^, ^17^

To denoise the data, residual movement and physiological noise were removed using a 0.008–0.09 Hz bandpass filter with linear detrending and an ART scrubbing protocol. Furthermore, the CompCor strategy, which involves removing physiological noise with anatomical component‐based noise correction, was applied to reduce physiological noise at the BOLD level.^18^

### 2.6 fMRI processing and connectivity analysis

In this study, Group ICA (Independent Component Analysis) was used to measure the connectivity change on each pair of ROIs.^4^ The first‐level analysis was performed on a voxel‐to‐voxel basis, summarizing the functional connectivity between each voxel. Local correlation analysis was used as a voxel‐to‐voxel measure of functional segregation for each observational point. The average correlation between the time courses in each seed voxel and its neighbors was used to measure local functional coupling for each voxel. A voxel neighborhood was defined as the probability area enclosed by an isotropic Gaussian kernel.

Group‐level independent component analyses (group‐ICA^19^) were performed to estimate 40 temporally coherent networks from the fMRI data combined across all subjects and conditions.^20^, ^21^ The BOLD signal from every timepoint and voxel in the brain was concatenated across subjects and conditions along the temporal dimension. A singular value decomposition of the z‐score normalized BOLD signal (subject‐level SVD) with 64 components separately for each subject, and the condition was used as a subject‐specific dimensionality reduction step. The dimensionality of the concatenated data was further reduced using a singular value decomposition (group‐level SVD) with 40 components, and a fast‐ICA fixed‐point algorithm^22^ with hyperbolic tangent (G1) contrast function was used to identify spatially independent group‐level networks from the resulting components. Finally, GICA3 back‐projection^10^ was used to compute ICA maps associated with these same networks separately for each individual subject and condition.

Group‐level analyses were performed using a General Linear Model (GLM^4^). For each individual voxel, a separate GLM was estimated, with first‐level connectivity measures at this voxel as dependent variables (one independent sample per subject and one measurement per task or experimental condition, if applicable), and groups or other subject‐level identifiers as independent variables. Voxel‐level hypotheses were evaluated using multivariate parametric statistics with random effects across subjects and sample covariance estimation across multiple measurements. Inferences were performed at the level of individual clusters (groups of contiguous voxels). Cluster‐level inferences were based on parametric statistics from Gaussian random field theory.^4^, ^23^ Results were thresholded using a combination of a cluster‐forming *p* < 0.001 voxel‐level threshold and a familywise corrected p‐FDR <0.05 cluster‐size threshold.^24^

The significance of these components on neural networks was defined by the spatial overlap of suprathreshold areas known as Dice Similarity Coefficiency or Sørensen‐Dice Index with visually selected best‐matched IC for each network.^4^, ^25^, ^26^

### 2.7 Statistical analyses

Statistical analyses were performed using SPSS 25 (SPSS Inc., Chicago, IL, USA). Data normality was evaluated and for group differences, Shapiro Wilk, Mann–Whitney *U*, and chi‐square tests were applied to available data.

All second‐level analyses were performed on the control>aMCI between‐subject contrast with age as a covariate [1, −1, 0], and any effects (F‐test) were applied at the between‐source contrast level with *p* < 0.05 (FDR: False Discovery Rate‐corrected; p‐uncorrected <0.001) with a minimum cluster size of 50 contiguous voxels.^27^

### 2.8 Ethical status

The University Ethics Committee approved research as number 280 on 29/03/2018.

## 3 RESULTS

**FIGURE 1:**
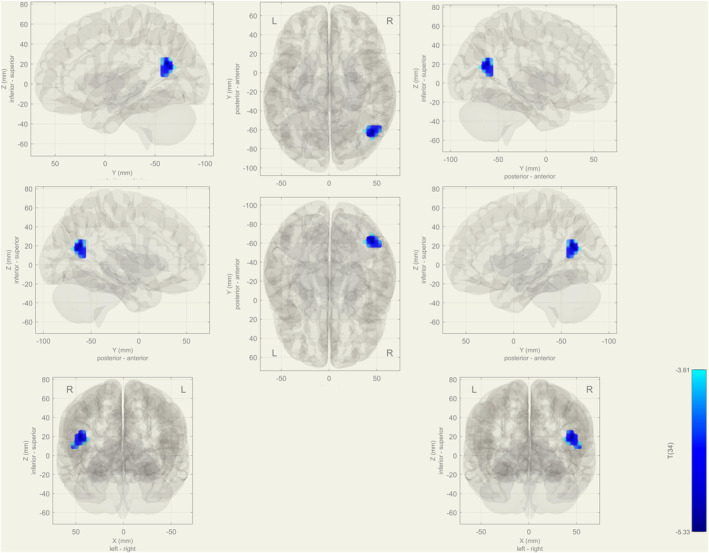
During rs‐ fMRI increasing functional connectivity was seen in the visual network in the aMCI group as a compensation mechanism at IC‐5 (represented visual network); in the right superior and inferior lateral occipital cortex, angular gyrus, and middle temporal gyrus (temporo‐occipital part).

**FIGURE 2:**
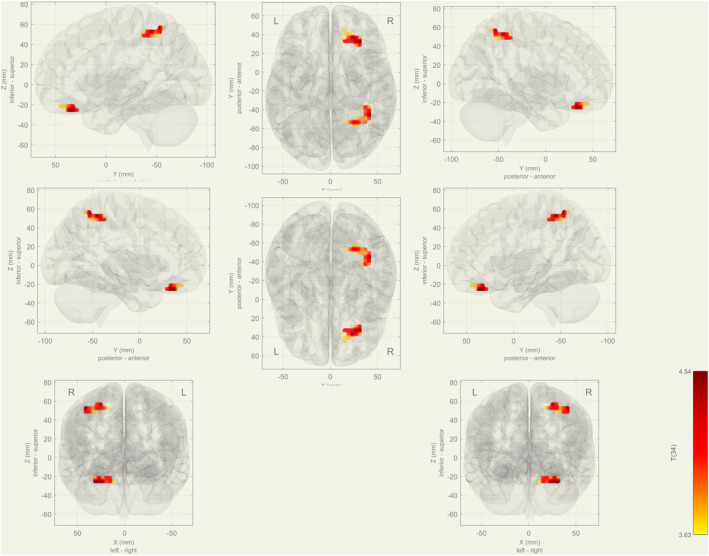
Bilateral thalamus and caudate nucleus functional connectivity decreased in the aMCI group during rs‐fMRI at IC‐36 (represented frontoparietal network).

In the rs‐fMRI, increasing activity was observed in the right superior and inferior lateral occipital cortex, the right angular gyrus, and the temporo‐occipital part of the right middle temporal gyrus at IC‐5 in the aMCI group compared with healthy controls (p‐FDR = 0.008) (Figure 1). At IC‐36, the bilateral thalamus and caudate nuclei showed decreased activity in the frontoparietal network in the aMCI group (p‐FDR = 0.002) (Figure 2 and Table 2).

**TABLE 2:** ICA rs‐fMRI results.

MNI coordinates (*x*, *y*, *z*)	Total voxel size	p‐FDR	Network	Area	ROI (%)	Voxel size
+46–62+20	296	0.008	IC‐5 (visual)	Right superior lateral occipital cortex	2	115
				Right inferior lateral occipital cortex	3	55
				Right angular gyrus	3	47
				Right middle temporal gyrus temporo‐occipital part	4	42
−10−06+08	496	0.001	IC‐36 (frontoparietal)	Left thalamus	7	98
				Right thalamus	7	91
				Right caudate	9	46
				Left caudate	7	36

*Note*: CONN uses Oxford‐Harvard ROI atlas as standard and matches it with MNI coordinates.

Abbreviations: FDR, False Discovery Rate; IC, Independent component; MNI, Montreal Neurological Institute; ROI, Region of interest.

**FIGURE 3:**
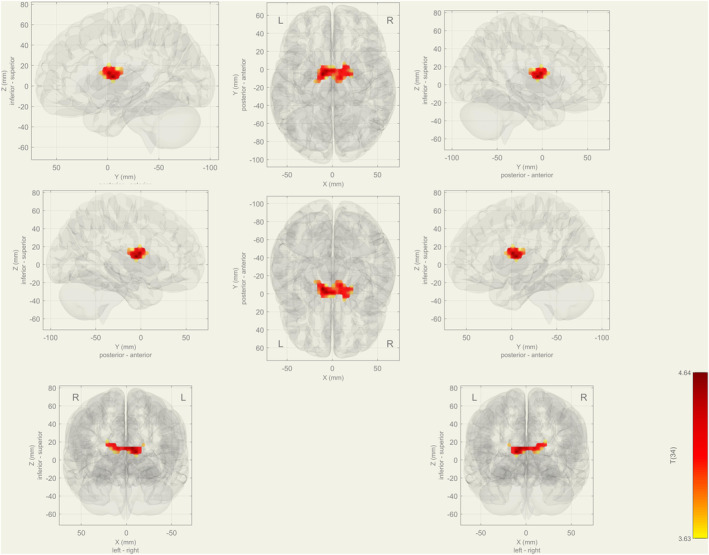
During the oddball task, decreased functional connectivity was seen within the visual network at the aMCI group in both left and right frontal pole, right frontal orbital cortex, left superior parietal lobule, right postcentral gyrus, right posterior part of supramarginal gyrus, right superior part of the lateral occipital cortex, and right angular gyrus.

In the oddball task, the right frontal pole, right frontal orbital cortex, left superior parietal lobule, right postcentral gyrus, right posterior part of supramarginal gyrus, right superior part of the lateral occipital cortex, and right angular gyrus showed a significant decrease in functional connectivity in the aMCI group compared to healthy controls (Figure 3) (Table 3).

**TABLE 3:** Visual Oddball Task ICA Results.

MNI coordinates (*x*, *y*, *z*)	Total voxel size	p‐FDR	Network	Area	ROI (%)	Voxel size
+42–46+52	248	0.014	IC‐20 (visual)	Left superior parietal lobule	14	203
+32+32–26	171	0.029		Right frontal pole	1	72
				Right frontal orbital cortex	2	22
				Right post central gyrus	0	13
				Right posterior supramarginal gyrus	1	9
				Right superior lateral occipital cortex	0	8
				Right angular gyrus	0	4

*Note*: CONN uses Oxford‐Harvard ROI atlas as standard and matches it with MNI coordinates.

Abbreviations: FDR, False Discovery Rate; IC, Independent component; MNI, Montreal Neurological Institute; ROI, Region of interest.

## 4 DISCUSSION

The present study aimed to investigate the early signs of amnestic mild cognitive impairment (aMCI) using independent component analysis (ICA) combined with the visual oddball task in a group of older adults. Our results demonstrated that this approach effectively identified changes in functional connectivity in individuals with aMCI compared to healthy controls.

ICA has been used in previous studies to identify distinct patterns of neural activity that are consistent across a group of individuals.^28^ In this study, we applied ICA to identify patterns of neural activity associated with the visual oddball task in both aMCI and control groups. Our results showed that aMCI patients exhibited decreased functional connectivity in regions associated with attention and executive function in the right middle temporal gyrus and frontal areas such as the frontal pole and the frontal orbital cortex. In the MCI group, MFG, IFG, PCG, SPL and bilateral thalamus showed decreased activity in rs‐fMRI, which is consistent with previous research and our results.^29^, ^30^, ^31^

The visual oddball task has been shown to be effective in identifying early signs of cognitive decline.^32^ By presenting a series of visual stimuli with occasional “oddball” stimuli, this task engages attention and working memory, which are cognitive functions that are commonly affected in aMCI. Our study supports previous research that applied EEG by demonstrating that the visual oddball task can be used in conjunction with ICA to detect early signs of cognitive decline in individuals with aMCI.^15^, ^33^

The current study found significant relationships between the thalamus and the frontoparietal network, and the caudate nucleus and the visual network in patients with aMCI using rs‐fMRI. These findings are consistent with previous literature that has linked reduced thalamic activity to various functions, such as mood, depression, and attention, in patients with aMCI.^31^, ^34^ The caudate nucleus has also been reported to be involved in various cognitive processes such as learning, memory, and attention. Also, right caudate atrophy was found to be related to aMCI> AD conversion at longitudinal studies.^35^, ^36^

Studies have indicated that there are differences in rs‐fMRI analysis results between AD and MCI, which may be due to differences in the patient groups and methodology used in the studies.^28^ Recent studies have also focused on the angular gyrus and frontal connections, in addition to the thalamus and hippocampus, in differentiating HC, MCI, and AD.^3^, ^36^, ^37^ The angular gyrus, frontal connections, and hippocampus have been identified as areas of interest in recent research on rs‐fMRI analysis in AD and MCI patients.^37^ In this study, the right superior lateral occipital cortex, right inferior lateral occipital cortex, right angular gyrus, and right middle temporal gyrus temporo‐occipital part were identified as regions of interest in the visual network. These regions have been implicated in visual processing, object recognition, and visual attention.^38^ The increased activity observed in the right superior lateral occipital cortex during resting state fMRI analysis in this study could be interpreted as a compensation mechanism for neuronal degeneration in AD and MCI patients, as reported in a previous study.^15^

The ICA results of the visual oddball task also revealed several areas of interest, including the left superior parietal lobe, the right frontal pole, the right frontal orbital cortex, the right postcentral gyrus, the right posterior supramarginal gyrus, the right superior lateral occipital cortex, and the right angular gyrus. These regions have previously been implicated in attention, working memory, and executive control processes.^39^ Interestingly, in this study, the changes observed in frontal activity during the oddball task, also alpha waves were found in the frontal and central areas to be specific for the oddball task in the EEG study.^33^

In our study, we investigated the role of specific brain regions in individuals with Mild Cognitive Impairment (MCI) during a task that measured disinhibition, reward processing, and information manipulation. We have seen decreased functional connectivity in these areas, and the visual network is strongly associated with frontoparietal areas during a visual cognitive task. We found that the MCI group exhibited decreased activity in the frontal pole, suggesting impaired disinhibition. In a visual task, reduced activity in the orbitofrontal cortex was observed, indicating deficits in reward‐based learning and recognition processes. Furthermore, decreased activity in the superior parietal lobule was associated with impaired information manipulation and visuospatial perception. These findings highlight the potential neurobiological mechanisms underlying the difficulties experienced by individuals with MCI in performing the task, providing insights for future interventions targeting cognitive deficits in this population.^40^, ^41^, ^42^, ^43^

EEG studies have shown that theta wave activity in the temporoparietal region is consistently increased in individuals with AD and MCI compared to healthy controls.^44^, ^45^ Theta waves are associated with cognitive processing and memory formation, and increased theta activity in the temporal lobe has been linked to episodic memory deficits in AD (21). In addition, studies have found that theta wave activity in the parietal cortex is associated with semantic processing deficits in AD.^46^

Structural MRI studies have also demonstrated atrophy in the temporoparietal region in individuals with AD and MCI.^47^, ^48^ The temporoparietal region is a critical brain area involved in memory and language processing, and its atrophy is consistent with the cognitive deficits observed in AD and MCI^3^.

In previous oddball EEG studies, higher frontal‐occipital gamma coherence and lower parieto‐occipital gamma coherence were observed in AD patients with AD who received treatment compared to those who did not receive treatment.^15^, ^49^ It is unclear whether this pattern of activity is a compensatory mechanism or is caused by damage to inhibitory neurons. The findings of the current study also reflect this fronto‐occipital connectivity, indicating that the oddball test can detect changes in these regions.

Studies using EEG and structural MRI have found that changes in the temporoparietal region, particularly in theta wave activity and atrophy, are distinguishing features of AD and MCI. In addition, alpha wave activity in the frontal and central areas has been found to be specific to the oddball test. These findings are consistent with previous studies that have observed changes in frontal activity during the oddball task and a decrease in temporoparietal connectivity and left temporal atrophy. The increased activity observed in the right superior lateral occipital cortex during resting‐state activity may indicate a compensatory mechanism for neuronal degeneration. This is supported by previous EEG studies that have found reduced activity during task‐based analysis.^15^, ^33^, ^49^, ^50^, ^51^

Our findings are consistent with previous studies that have identified altered connectivity in these brain areas in both MCI and AD.^52^, ^53^ Our study supports these studies by demonstrating these changes in connectivity may occur early in the disease process, potentially serving as markers of cognitive decline.

In summary, the findings of the current study are consistent with previous research on resting‐state fMRI, EEG, and structural MRI in AD and MCI. This research is important because it is the first in the literature to use a visual oddball task on fMRI in patients with aMCI. These findings suggest that the oddball test may be a useful tool for identifying early changes in brain activity in patients with aMCI. However, further research is needed to confirm these findings and determine the clinical utility of the oddball test in the diagnosis and treatment of aMCI and AD.

## 5 LIMITATIONS

While our findings are promising, there are several limitations to our study. First, our sample size was relatively small, which may limit the generalizability of our findings. Second, our study was cross‐sectional, which means that we cannot establish a causal relationship between changes in functional connectivity and the development of MCI. Longitudinal studies are needed to establish the temporal sequence of these changes and assess their predictive value for cognitive decline.

Despite these limitations, our study highlights the potential of ICA combined with the visual oddball task as a tool for detecting early signs of aMCI. This approach could be used in future studies to identify people at risk of developing cognitive decline and to evaluate the efficacy of interventions aimed at preventing or preventing the onset of dementia.

## 6 CONCLUSION

Our study provides evidence that ICA combined with the visual oddball task can effectively identify changes in functional connectivity associated with aMCI. Future studies with larger sample sizes and longitudinal designs are needed to further validate this approach and investigate its potential as a diagnostic tool for cognitive decline.

## FUNDING INFORMATION

This project was funded by the Scientific Research Projects Coordination Unit of the University with number 2014–154.

## CONFLICT OF INTEREST STATEMENT

The authors declare that they have no financial or personal relationships that may have influenced the work presented in this manuscript. They have no conflict of interest to disclose.

## Supporting information


Appendix S1

## Data Availability

The data that support the findings of this study are available on request from the corresponding author. The data are not publicly available due to privacy or ethical restrictions.
