# Supplementary material for: Functional magnetic resonance imaging study during resting state and visual oddball task in mild cognitive impairment
Source: CNS Neurosci Ther. 2023 Jul 20;30(2):e14371. doi: 10.1111/cns.14371 (PMC10848090; doi:10.1111/cns.14371)
Supplement: Supplementary file 1 — Appendix S1 [file CNS-30-e14371-s001.pdf]

Tests of Normality

	Kolmogorov-Smirnov[a]			Shapiro-Wilk		
	Statistic	df	Sig.	Statistic	df	Sig.
Age	,095	37	,200*	,960	37	,209
Education	,140	37	,063	,944	37	,061

*. This is a lower bound of the true significance.

a. Lilliefors Significance Correction

Table 1. Test of Normality results

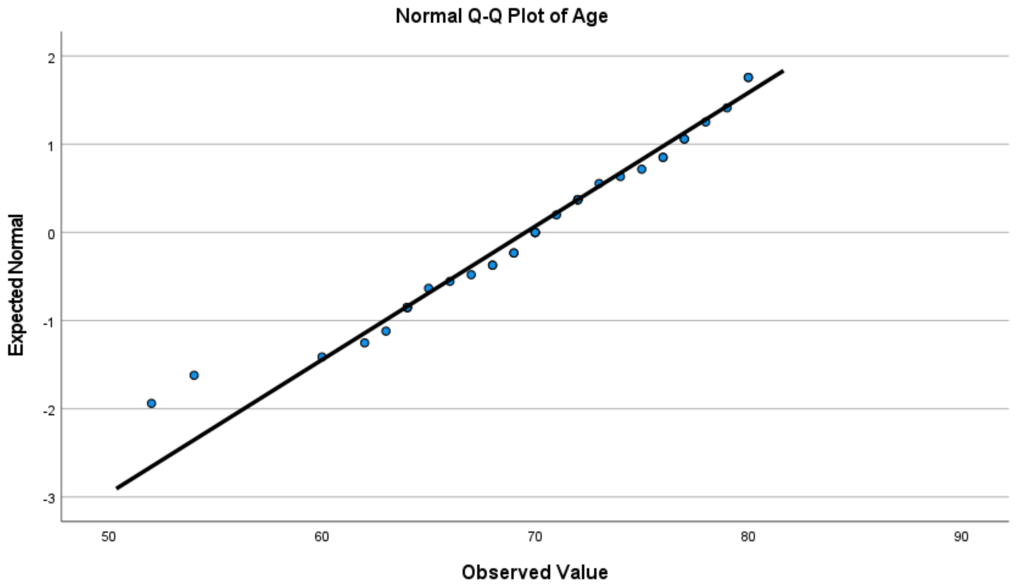

Figure 1. Normality of age derivation

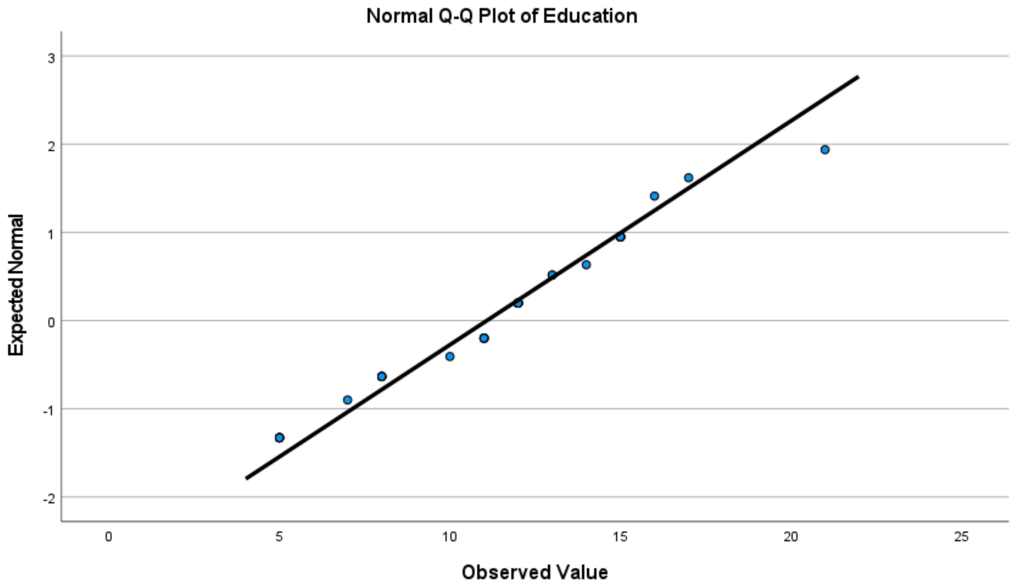

Figure 2. Normality of education (year) derivation

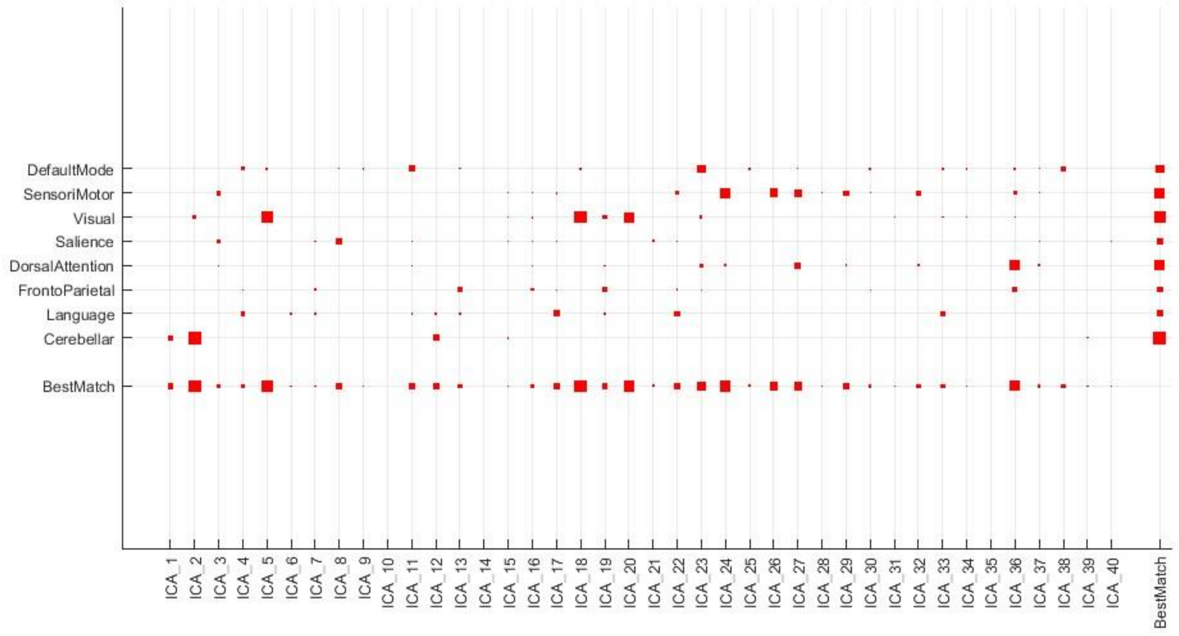

Figure 3. Dice Coefficiency Table of the Independent Components

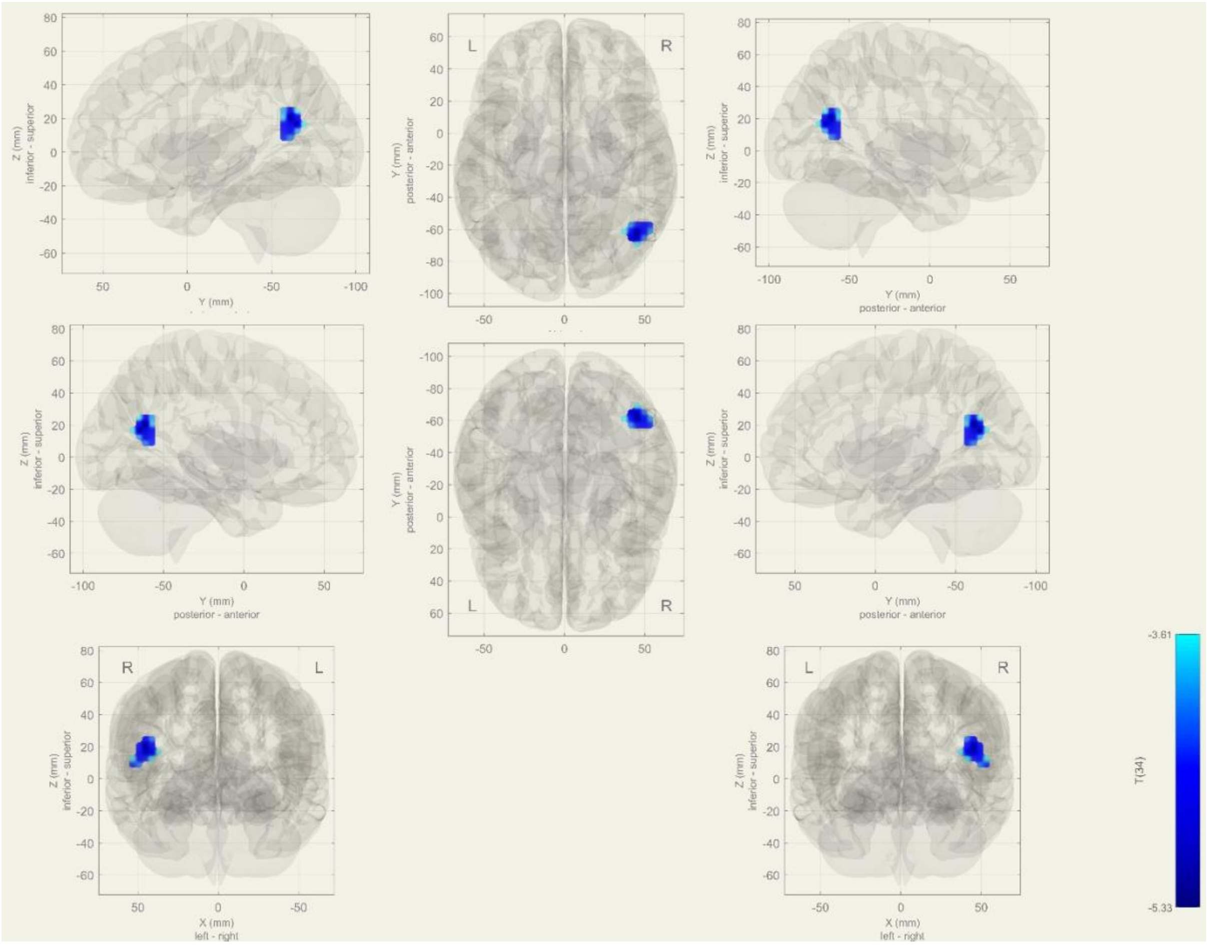

Figure 4. During rs- fMRI increasing functional connectivity was seen in the visual network in the aMCI group as a compensation mechanism at IC-5(represented visual network); in the right superior and inferior lateral occipital cortex, angular gyrus, and middle temporal gyrus (temporooccipital part).

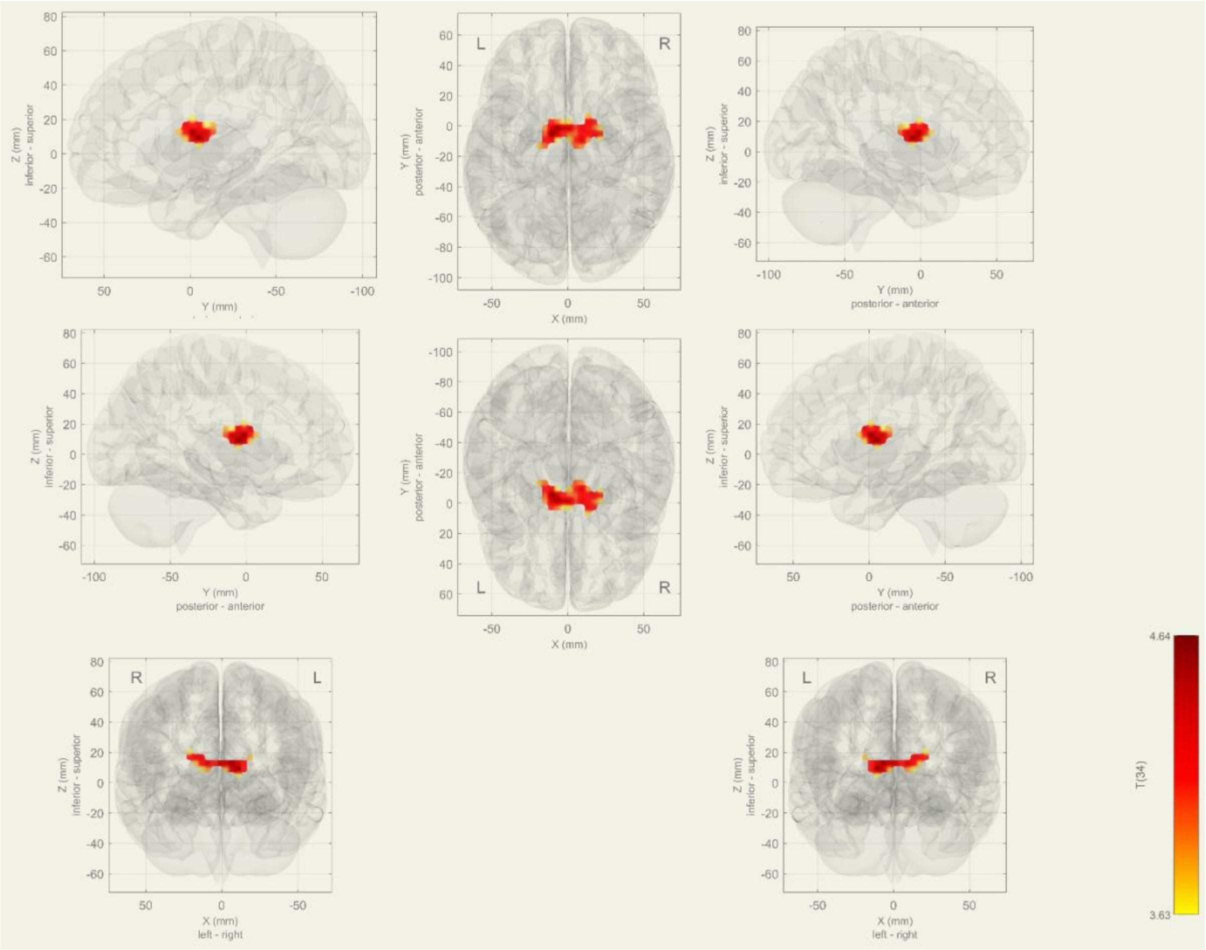

Figure 5. Bilateral thalamus and caudate nucleus functional connectivity decreased in the aMCI group during rs-fMRI at IC-36 (represented frontoparietal network).

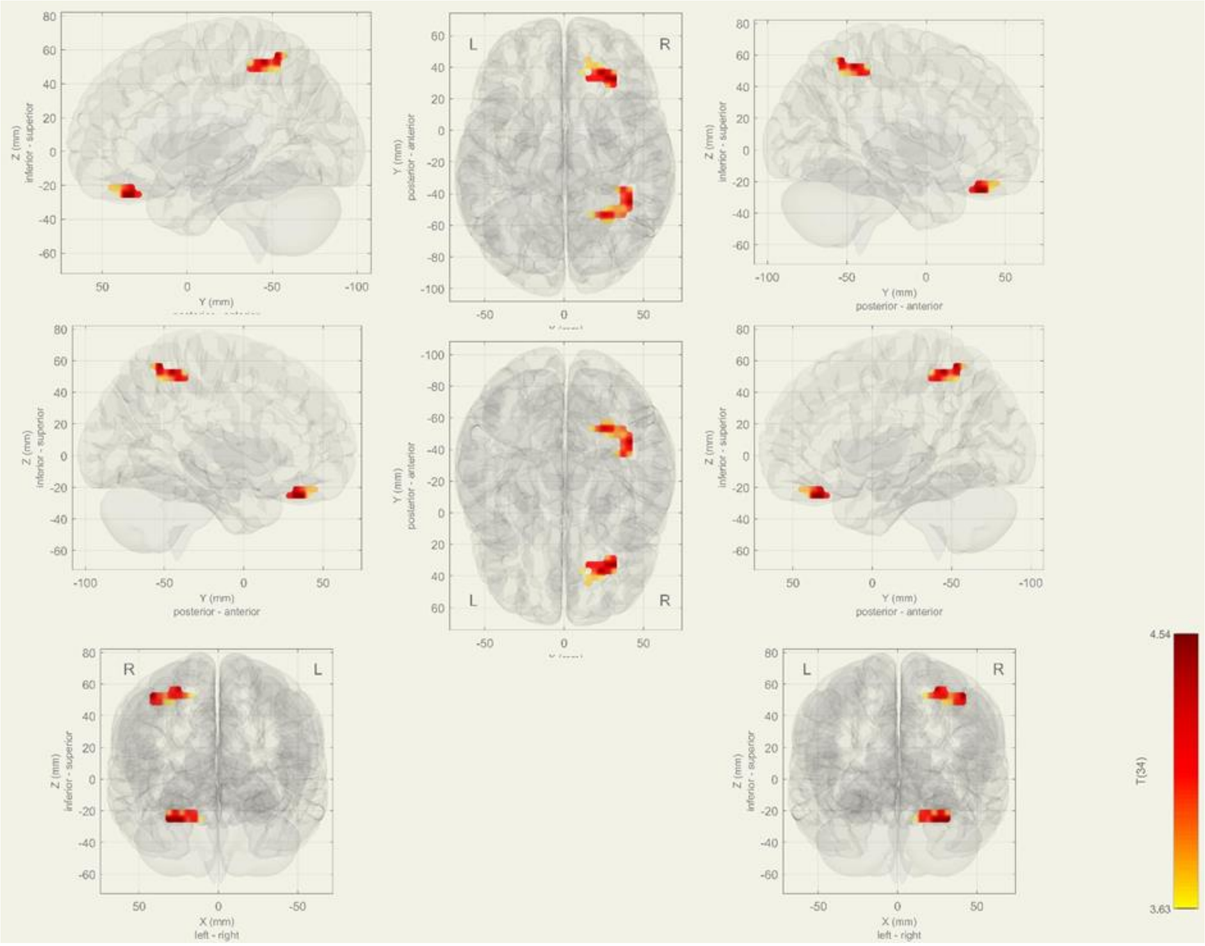

Figure 6. During the oddball task, decreased functional connectivity was seen within the visual network at the aMCI group in both left and right frontal pole, right frontal orbital cortex, left superior parietal lobule, right postcentral gyrus, right posterior part of supramarginal gyrus, right superior part of the lateral occipital cortex, and right angular gyrus.
